# Large language model–assisted support among caregivers of patients with epilepsy: associations with caregiver burden and psychosocial outcomes

**DOI:** 10.3389/fneur.2026.1912114

**Published:** 2026-09-09

**Authors:** Lan Lin, Ruixue Gao, Wanhui Dong

**Affiliations:** Department of Neurology, The First Bethune Hospital of Jilin University, Changchun, Jilin, China

**Keywords:** caregiver burden, DeepSeek, epilepsy, large language model, psychosocial outcomes

## Abstract

**Objective:**

To investigate the association between large language model (LLM)-assisted support and caregiver burden as well as psychosocial outcomes among family caregivers of patients with epilepsy.

**Methods:**

This single-center, prospective cohort study enrolled primary caregivers of patients with epilepsy. Participants were classified into an LLM group and a control group based on whether they had used DeepSeek for epilepsy caregiving-related support. Caregiver burden was assessed using the Zarit Burden Interview (ZBI). Secondary outcomes included anxiety, depression, perceived stress, social support, quality of life, and caregiving self-efficacy, measured at baseline, 1 month, and 3 months. Adjusted generalized estimating equation (GEE) models were used to evaluate longitudinal associations between LLM use and the study outcomes, and dose-response analyses were performed within the LLM group.

**Results:**

A total of 296 caregivers were included (149 in the control group and 147 in the LLM group). The two groups were comparable at baseline. At 3 months, the LLM group had lower caregiver burden (median ZBI: 23.00 vs. 25.00), anxiety, depression, and perceived stress scores, as well as a higher perceived social support score, than the control group. Adjusted GEE analyses revealed significant between-group differences in changes in caregiver burden and depression at both follow-up assessments and in anxiety, perceived stress, and psychological quality of life at 3 months, favoring the LLM group. Within the LLM group, no significant dose–response associations were observed between weekly usage time or the weekly number of effective conversations and caregiver burden at 3 months.

**Conclusions:**

Among caregivers of patients with epilepsy, use of LLM-assisted support was associated with lower caregiver burden and better psychosocial outcomes over 3 months. LLMs may serve as a low-threshold, scalable supportive tool in epilepsy family caregiving, though further studies are needed to evaluate long-term effectiveness and safety.

## 1 Introduction

Epilepsy is a common neurological disorder affecting approximately 70 million people worldwide, with an estimated 2.4 million new cases diagnosed each year (1). As a chronic condition characterized by recurrent seizures, epilepsy is marked by the sudden and unpredictable nature of seizure events, which not only compromise patients' physical and psychological health but also interfere with daily life, work, and overall quality of life (2). For family caregivers, caregiving is not a single task but an ongoing and multifaceted responsibility that includes seizure monitoring and response, medication management, coordination of medical care, assistance with daily activities, and emotional support (3). This long-term, dynamic, and highly demanding caregiving process often places caregivers under substantial physical and psychological strain, further affecting anxiety, depression, perceived stress, and quality of life (4).

Caregiver burden in epilepsy has been investigated in a growing body of literature. Survey-based and clinical studies have shown that anxiety, depression, and other emotional problems are common among caregivers of patients with epilepsy, and that caregiver burden is closely related to disease characteristics, caregiving demands, and family resources (5). At the same time, interventions aimed at reducing caregiver burden have increasingly been explored, including family empowerment, psychological support, health education, and caregiver training (6). However, important gaps remain in existing support pathways. On the one hand, epilepsy-related informational and service needs are often insufficiently met, and caregivers frequently face difficulties in accessing medical information and obtaining adequate knowledge support (7). On the other hand, even when online information is available, its readability and comprehensibility are often suboptimal, limiting its translation into practical and high-quality caregiving guidance (8). In addition, traditional support models still rely heavily on face-to-face consultations and relatively complex healthcare processes, which may restrict timely access to help because of time, geographic, and cost barriers, and may be insufficient to meet caregivers' dual needs for immediate problem solving and emotional support (9).

Recent advances in large language models (LLMs) may offer a new approach to addressing these challenges. LLMs can understand and generate natural language and have been applied in healthcare for information summarization, medical question answering, information collection, and communication support (10). In caregiving settings, they may help provide tailored explanations, integrate information, and offer supportive feedback, thereby improving access to knowledge, enhancing self-efficacy, and potentially reducing caregiver burden. Emerging evidence suggests that LLMs show promise in family caregiving support, although their effectiveness and reliability require further validation in specific disease contexts (11).

Against this background, the present study aimed to investigate the potential value of LLMs in reducing caregiver burden among caregivers of patients with epilepsy. We compared epilepsy caregivers who used an LLM-based support tool (DeepSeek) with those who did not in terms of caregiver burden and psychosocial outcomes, and further evaluated the independent associations between LLM use and caregiver burden, anxiety, depression, and related outcomes. To this end, we conducted a prospective cohort study in which participants were grouped according to whether they used DeepSeek to obtain epilepsy caregiving-related support, and were followed up at 1 month and 3 months after baseline to evaluate the short- and medium-term effects of LLM use on caregiver burden and related psychosocial outcomes. Through adjusted longitudinal analyses, this study also sought to evaluate the associations between LLM use and caregiver burden and related psychosocial outcomes in real-world caregiving settings.

## 2 Methods

### 2.1 Study design and ethical approval

This was a single-center, prospective cohort study reported in accordance with the STROBE statement. The study was conducted in the Department of Neurology at a tertiary hospital. Primary caregivers of patients with epilepsy were enrolled at baseline and followed up at 1 month and 3 months. Participants were classified into the LLM group or the control group based on whether they had used DeepSeek for epilepsy caregiving-related support. The study was approved by the Ethics Committee of The First Bethune Hospital of Jilin University (Approval No. K2026154) and conducted in accordance with the Declaration of Helsinki. Written informed consent was obtained from all participants.

### 2.2 Participants

Participants were primary informal caregivers of patients with epilepsy, including spouses, parents, children, and other family members with long-term caregiving responsibilities. Paid or professional caregivers were excluded. They were mainly recruited from the neurology outpatient clinic, epilepsy clinic, and inpatient wards, with additional recruitment through the hospital follow-up system or patient communities. For those recruited outside the hospital, the diagnosis of epilepsy and caregiver status were further verified.

Inclusion criteria were: age ≥20 years; being the primary unpaid informal caregiver of a clinically diagnosed patient with epilepsy for at least 3 months; having major caregiving responsibility during the study period; being able to understand and complete questionnaires independently; providing written informed consent; and completing the baseline assessments. Exclusion criteria were: severe psychiatric disorders, marked cognitive impairment, or other conditions affecting study participation; current intensive psychotherapy, psychiatric hospitalization, or other strong interventions that might substantially affect psychosocial outcomes; inability to complete the baseline survey or expected inability to complete follow-up; being a paid or professional caregiver; not being the patient's primary caregiver; or having an unstable caregiving relationship.

The sample size estimation was based on pilot study data, which showed that the average Zarit Burden Interview (ZBI) score in the LLM group was 23.75 and in the control group was 27. With 80% statistical power, a two-sided α level of 0.05, and an anticipated dropout rate of 20%, the required sample size was calculated to be 308 participants.

### 2.3 Exposure

The main exposure was DeepSeek-v3.2 use. Participants who had used DeepSeek for epilepsy caregiving-related support were classified into the LLM group, and those who had not were classified into the control group. We also collected dose-related measures, including days of use per week, and number of effective epilepsy caregiving-related conversations per week. When available, platform logs were used; otherwise, structured self-report was used.

### 2.4 Outcomes

The primary outcome was caregiver burden, measured by the 22-item Zarit Burden Interview (ZBI), with higher scores indicating greater burden (12). Secondary outcomes included anxiety, depression, perceived stress, social support, quality of life, and caregiving self-efficacy. Anxiety and depression were measured using the Hospital Anxiety and Depression Scale (HADS-A and HADS-D), perceived stress using the Perceived Stress Scale-10 (PSS-10), social support using the Multidimensional Scale of Perceived Social Support (MSPSS), and quality of life using the WHOQOL-BREF. Caregiving self-efficacy was measured using the total self-efficacy score (13–17). All outcomes were assessed at baseline (T0), 1 month (T1), and 3 months (T2).

### 2.5 Covariates

Potential confounders were also collected. Caregiver-related variables included age, sex, marital status, education, duration of caregiving, daily caregiving time, other caregiving support, sleep status, and mental health history. Patient-related variables included age, duration of epilepsy, seizure frequency during the previous 3 months, number of antiseizure medications, burden of adverse effects, and drug-resistant epilepsy. These variables were collected at baseline.

### 2.6 Data collection and follow-up

At baseline, researchers collected demographic and clinical information and administered the ZBI, HADS, PSS-10, MSPSS, WHOQOL-BREF, and caregiving self-efficacy assessments. The same outcomes were reassessed at 1 month and 3 months, and DeepSeek use during follow-up was also recorded. Standardized procedures, staff training, logic checks, and outlier screening were used to improve data quality. DeepSeek use recorded during follow-up was mainly used for descriptive analyses and dose-response analyses within the LLM group, whereas the main group comparisons and longitudinal models were based on baseline exposure group.

### 2.7 Statistical analysis

The Shapiro-Wilk test was used to assess normality. Continuous variables are presented as mean ± standard deviation or median (interquartile range), and categorical variables as counts and percentages. Between-group comparisons were performed using the independent-samples *t* test or Mann-Whitney *U* test for continuous variables, and the chi-square test or Fisher's exact test for categorical variables.

Outcomes at each time point and their raw changes from baseline were summarized descriptively. Because repeated measurements were obtained from the same caregivers, adjusted generalized estimating equation (GEE) models were used to evaluate longitudinal associations between LLM use and the study outcomes. The models included group, time, group-by-time interaction terms, and the prespecified baseline covariates described above, with the control group and T0 as the reference categories. A Gaussian distribution with an identity link function and an exchangeable working correlation structure was specified. Robust sandwich variance estimators were used to calculate standard errors, 95% confidence intervals, and *P* values. Post-estimation linear contrasts were used to estimate the mean changes from baseline at T1 and T2 within each group and the between-group differences in mean change. Because all participants completed the three assessments, no outcome data were missing and no imputation was performed. In addition, dose-response analyses were performed within the LLM group to explore the associations of weekly use time and weekly number of effective conversations with caregiver burden at 3 months. All tests were two-sided, and P < 0.05 was considered statistically significant. All analyses were performed using R version 4.3.2.

## 3 Results

### 3.1 Baseline characteristics

**Figure 1:**
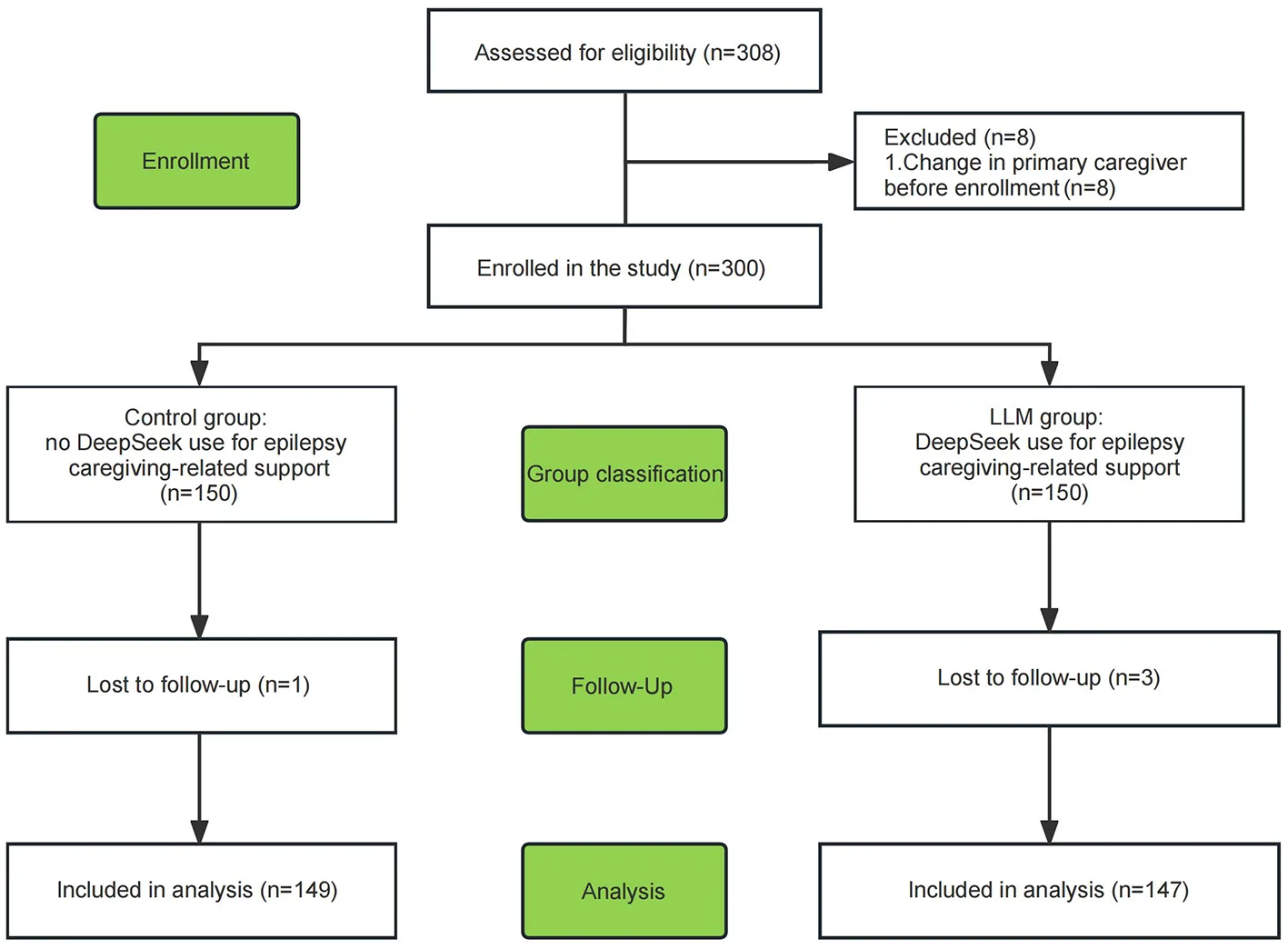
Study Flowchart. LLM = large language model.

A total of 296 caregivers of patients with epilepsy were included, comprising 149 in the control group and 147 in the LLM group (Figure 1). All participants completed the baseline, 1-month, and 3-month assessments, yielding 888 complete longitudinal observations. The two groups were generally comparable at baseline. The mean age of the overall sample was 45.17 ± 11.52 years; the mean ages were 45.28 ± 11.56 years in the control group and 45.05 ± 11.52 years in the LLM group, with no significant between-group difference. The median duration of caregiving was 34.00 (21.00, 47.00) months in the control group and 37.00 (26.00, 48.00) months in the LLM group. Daily caregiving time was 7.34 ± 2.23 h and 7.17 ± 2.21 h, respectively, with no significant difference between groups. Patient-related characteristics, including age, duration of epilepsy, seizure frequency during the previous 3 months, number of antiseizure medications, burden of adverse effects, and proportion of drug-resistant epilepsy, were also comparable between groups. The distributions of sex, marital status, and educational level were similar in the two groups (Table 1). The distributions of severity and interpretive categories for ZBI, HADS-A, HADS-D, and MSPSS at each assessment are presented in Supplementary Table S1.

**Table 1:** Baseline characteristics.

Variable	Overall (*N* = 296)	Control group (*n* = 149)	LLM group (*n* = 147)	*P* value
Caregiver age, years	45.17 ± 11.52	45.28 ± 11.56	45.05 ± 11.52	0.865
Duration of caregiving, months	35.00 (23.00, 48.00)	34.00 (21.00, 47.00)	37.00 (26.00, 48.00)	0.153
Daily caregiving time, hours	7.25 ± 2.21	7.34 ± 2.23	7.17 ± 2.21	0.511
Sleep status score (1–5)	3.00 (2.00, 4.00)	3.00 (3.00, 4.00)	3.00 (2.00, 4.00)	0.141
Patient age, years	24.00 (15.00, 33.00)	24.00 (15.00, 33.00)	24.00 (15.00, 33.50)	0.788
Duration of epilepsy, years	6.00 (4.00, 10.00)	6.00 (4.00, 10.00)	6.00 (3.00, 10.00)	0.475
Seizure frequency during the previous 3 months	7.00 (4.00, 12.00)	7.00 (4.00, 12.00)	8.00 (4.50, 11.00)	0.662
Number of antiseizure medications	2.00 (2.00, 3.00)	2.00 (2.00, 3.00)	2.00 (2.00, 3.00)	0.877
Burden of adverse effects (0–10)	5.00 (3.00, 6.00)	5.00 (3.00, 6.00)	4.00 (3.00, 6.00)	0.962
Female caregiver	205 (69.3%)	99 (66.4%)	106 (72.1%)	0.291
Married	159 (53.7%)	75 (50.3%)	84 (57.1%)	0.240
College degree or above	138 (46.6%)	67 (45.0%)	71 (48.3%)	0.565
Employed	149 (50.3%)	77 (51.7%)	72 (49.0%)	0.642
Middle-to-high income	175 (59.1%)	86 (57.7%)	89 (60.5%)	0.621
Spousal caregiver	98 (33.1%)	42 (28.2%)	56 (38.1%)	0.070
Living with the patient	245 (82.8%)	118 (79.2%)	127 (86.4%)	0.101
Other caregiving support	173 (58.4%)	88 (59.1%)	85 (57.8%)	0.829
Mental health history	66 (22.3%)	33 (22.1%)	33 (22.4%)	0.950
Drug-resistant epilepsy	106 (35.8%)	50 (33.6%)	56 (38.1%)	0.416

Data are presented as mean (SD), median (IQR), or *n* (%), as appropriate.

LLM, large language model.

### 3.2 Primary outcome

For the primary outcome, caregiver burden was similar between groups at baseline. The mean (SD) ZBI total scores were 30.70 (8.34) in the control group and 30.61 (8.81) in the LLM group. At 1 month, the mean (SD) ZBI scores were 26.77 (8.64) in the control group and 24.58 (8.91) in the LLM group. At 3 months, the corresponding scores were 24.77 (8.87) and 22.43 (8.12), respectively. From baseline to 1 month, the mean reductions in ZBI score were 3.94 (7.35) in the control group and 6.03 (7.50) in the LLM group. From baseline to 3 months, the corresponding reductions were 5.93 (7.16) and 8.18 (7.70), respectively, showing a numerically greater reduction in caregiver burden in the LLM group (Table 2).

**Table 2:** Primary and secondary outcomes.

Outcome	Time point	Control group (***n*** = 149)		LLM group (***n*** = 147)	
		Mean (SD)	Median (IQR)	Mean (SD)	Median (IQR)
ZBI total score	T0 baseline	30.70 (8.34)	31.00 (24.00, 36.00)	30.61 (8.81)	31.00 (25.00, 37.00)
	T1 1 month	26.77 (8.64)	26.00 (21.00, 34.00)	24.58 (8.91)	24.00 (17.00, 31.00)
	T2 3 months	24.77 (8.87)	25.00 (18.00, 32.00)	22.43 (8.12)	23.00 (15.00, 29.00)
	Change T1–T0	−3.94 (7.35)	−4.00 (-9.00, 1.00)	−6.03 (7.50)	−6.00 (-11.00,−2.00)
	Change T2–T0	−5.93 (7.16)	−6.00 (-11.00,−1.00)	−8.18 (7.70)	−8.00 (-14.00,−3.00)
HADS-A (anxiety)	T0 baseline	6.69 (2.21)	7.00 (5.00, 8.00)	6.56 (2.00)	6.00 (5.00, 8.00)
	T1 1 month	6.31 (2.21)	6.00 (5.00, 8.00)	5.93 (2.29)	6.00 (5.00, 7.00)
	T2 3 months	5.82 (2.16)	6.00 (4.00, 7.00)	5.10 (2.30)	5.00 (4.00, 7.00)
	Change T1–T0	−0.38 (2.58)	−1.00 (-2.00, 1.00)	−0.63 (2.59)	0.00 (-2.00, 1.00)
	Change T2–T0	−0.87 (2.54)	−1.00 (-2.00, 1.00)	−1.46 (2.35)	−1.00 (-3.00, 0.00)
HADS-D (depression)	T0 baseline	5.74 (2.13)	6.00 (4.00, 7.00)	5.73 (1.99)	6.00 (4.00, 7.00)
	T1 1 month	5.09 (1.86)	5.00 (4.00, 6.00)	4.37 (2.02)	4.00 (3.00, 6.00)
	T2 3 months	5.00 (1.97)	5.00 (4.00, 7.00)	4.41 (1.75)	4.00 (3.00, 5.00)
	Change T1–T0	−0.64 (2.64)	−1.00 (-3.00, 1.00)	−1.35 (2.26)	−1.00 (-3.00, 0.00)
	Change T2–T0	−0.74 (2.41)	−1.00 (-3.00, 1.00)	−1.31 (2.20)	−2.00 (-3.00, 0.00)
PSS-10 (perceived stress)	T0 baseline	14.19 (4.04)	14.00 (11.00, 17.00)	14.42 (3.81)	14.00 (12.00, 16.00)
	T1 1 month	13.27 (4.13)	13.00 (11.00, 16.00)	12.45 (4.18)	12.00 (9.00, 15.00)
	T2 3 months	12.79 (4.02)	12.00 (10.00, 16.00)	11.77 (3.76)	11.00 (9.00, 15.00)
	Change T1–T0	−0.93 (4.91)	−1.00 (-4.00, 2.00)	−1.97 (4.68)	−2.00 (-5.00, 1.00)
	Change T2–T0	−1.40 (4.67)	−2.00 (-4.00, 2.00)	−2.65 (4.63)	−2.00 (-6.00, 0.00)
MSPSS (social support)	T0 baseline	50.96 (6.16)	51.00 (46.00, 56.00)	50.88 (5.71)	51.00 (47.00, 54.00)
	T1 1 month	51.09 (5.85)	51.00 (47.00, 55.00)	52.02 (5.58)	52.00 (49.00, 55.00)
	T2 3 months	51.48 (5.50)	51.00 (48.00, 55.00)	52.76 (5.48)	53.00 (49.00, 57.00)
	Change T1–T0	0.13 (7.90)	−1.00 (-6.00, 5.00)	1.14 (7.52)	1.00 (-4.00, 6.50)
	Change T2–T0	0.52 (7.57)	0.00 (-5.00, 5.00)	1.88 (6.74)	2.00 (-2.00, 6.00)
Caregiving self-efficacy	T0 baseline	20.52 (2.95)	20.00 (18.00, 23.00)	21.05 (2.65)	21.00 (19.00, 23.00)
	T1 1 month	22.79 (3.11)	23.00 (20.00, 25.00)	24.01 (3.15)	24.00 (22.00, 26.00)
	T2 3 months	24.72 (3.29)	25.00 (23.00, 27.00)	25.94 (3.80)	25.00 (24.00, 28.00)
	Change T1–T0	2.27 (4.41)	2.00 (-1.00, 6.00)	2.97 (3.61)	3.00 (1.00, 5.00)
	Change T2–T0	4.19 (4.34)	4.00 (1.00, 7.00)	4.89 (4.44)	5.00 (2.00, 8.00)
WHOQOL physical domain	T0 baseline	47.32 (13.44)	46.67 (40.95, 58.11)	47.80 (13.33)	46.53 (40.30, 58.99)
	T1 1 month	52.17 (14.40)	50.45 (43.89, 63.58)	55.30 (14.62)	55.55 (43.44, 61.60)
	T2 3 months	56.14 (14.86)	52.52 (45.60, 66.35)	58.60 (14.83)	59.87 (45.49, 67.06)
	Change T1–T0	4.85 (19.56)	6.98 (-10.18, 16.91)	7.50 (17.63)	8.48 (-3.36, 21.12)
	Change T2–T0	8.82 (19.94)	8.24 (-2.26, 20.88)	10.80 (19.31)	12.39 (-1.03, 25.33)
WHOQOL psychological domain	T0 baseline	56.30 (15.16)	54.98 (49.01, 66.92)	55.66 (14.56)	55.55 (44.69, 66.41)
	T1 1 month	60.43 (14.07)	62.39 (51.43, 67.87)	63.50 (14.71)	66.88 (55.31, 72.67)
	T2 3 months	62.74 (14.62)	62.67 (51.81, 73.53)	67.90 (15.39)	66.70 (53.58, 79.81)
	Change T1–T0	4.13 (17.83)	2.91 (-8.54, 16.91)	7.84 (19.98)	9.92 (-5.49, 21.85)
	Change T2–T0	6.44 (17.82)	6.61 (-7.52, 19.09)	12.24 (19.12)	10.02 (-0.84, 25.39)
WHOQOL social domain	T0 baseline	70.09 (14.45)	70.42 (61.40, 79.45)	71.38 (15.91)	68.89 (57.59, 80.20)
	T1 1 month	72.16 (14.07)	70.34 (61.07, 79.62)	74.35 (14.22)	77.55 (68.21, 86.90)
	T2 3 months	71.84 (14.03)	70.05 (60.21, 79.89)	73.48 (13.73)	70.18 (60.03, 80.34)
	Change T1–T0	2.07 (19.69)	0.17 (-9.60, 18.22)	2.97 (22.21)	3.23 (-12.00, 18.00)
	Change T2–T0	1.75 (18.88)	0.44 (-10.22, 11.10)	2.10 (22.16)	1.29 (-10.02, 20.70)
WHOQOL environmental domain	T0 baseline	65.95 (10.48)	65.34 (58.88, 71.81)	65.24 (10.65)	65.08 (58.94, 71.23)
	T1 1 month	66.77 (10.31)	67.61 (59.24, 73.20)	68.80 (10.98)	71.81 (62.26, 78.18)
	T2 3 months	68.61 (10.22)	69.41 (60.85, 75.13)	70.90 (10.54)	69.95 (62.94, 76.95)
	Change T1–T0	0.82 (12.66)	1.83 (-7.86, 10.05)	3.56 (12.86)	3.76 (-5.78, 12.98)
	Change T2–T0	2.66 (13.05)	2.19 (-5.25, 9.78)	5.66 (13.79)	4.43 (-1.71, 14.73)

Data are presented as mean (SD) and median (IQR).

ZBI, zarit burden interview; HADS-A, hospital anxiety and depression scale-anxiety subscale; HADS-D, hospital anxiety and depression scale-depression subscale; PSS-10, 10-item perceived stress scale; MSPSS, multidimensional scale of perceived social support; WHOQOL, world health organization quality of life.

### 3.3 Secondary outcomes

For the secondary outcomes, anxiety, depression, perceived stress, and perceived social support were generally similar between groups at baseline. During follow-up, the LLM group showed a more favorable numerical pattern. At 3 months, the mean (SD) HADS-A scores were 5.82 (2.16) in the control group and 5.10 (2.30) in the LLM group; the mean (SD) HADS-D scores were 5.00 (1.97) and 4.41 (1.75), respectively; and the mean (SD) PSS-10 scores were 12.79 (4.02) and 11.77 (3.76), respectively. The mean (SD) MSPSS total scores were 51.48 (5.50) in the control group and 52.76 (5.48) in the LLM group. Numerically higher scores in caregiving self-efficacy and several WHOQOL-BREF domains were also observed in the LLM group at follow-up (Table 2).

### 3.4 Longitudinal GEE analysis

Adjusted GEE models showed no significant baseline differences between the LLM and control groups for any outcome. For the ZBI total score, significant reductions from baseline were observed in both groups, with greater reductions in the LLM group than in the control group at 1 month (adjusted difference in mean change, −2.09; 95% CI, −3.77 to −0.40; *P* = 0.015) and 3 months (−2.24; 95% CI, −3.93 to −0.56; *P* = 0.009). Greater reductions in HADS-A scores were observed in the LLM group at 3 months, whereas greater reductions in HADS-D scores were observed at both 1 and 3 months. At 3 months, the LLM group also showed greater improvements in PSS-10 scores and the psychological domain of the WHOQOL-BREF. No significant between-group differences in mean change were observed for MSPSS, caregiving self-efficacy, or the physical, social, and environmental domains of the WHOQOL-BREF (Table 3).

**Table 3:** Adjusted generalized estimating equation models for longitudinal outcomes.

Outcome	Clinically interpretable parameter	βadj (95% CI)	*P* value
ZBI total score	Baseline difference between groups (LLM vs Control)	−0.32 (-1.84, 1.19)	0.676
	Mean change at T1–T0 (Control group)	−3.94 (-5.12,−2.76)	< 0.001
	Mean change at T2–T0 (Control group)	−5.93 (-7.08,−4.79)	< 0.001
	Mean change at T1–T0 (LLM group)	−6.03 (-7.24,−4.82)	< 0.001
	Mean change at T2–T0 (LLM group)	−8.18 (-9.42,−6.94)	< 0.001
	Difference in mean change at T1–T0 (LLM vs Control)	−2.09 (-3.77,−0.40)	0.015
	Difference in mean change at T2–T0 (LLM vs Control)	−2.24 (-3.93,−0.56)	0.009
HADS-A	Baseline difference between groups (LLM vs Control)	−0.25 (-0.64, 0.13)	0.198
	Mean change at T1–T0 (Control group)	−0.38 (-0.80, 0.03)	0.069
	Mean change at T2–T0 (Control group)	−0.87 (-1.28,−0.47)	< 0.001
	Mean change at T1–T0 (LLM group)	−0.63 (-1.05,−0.21)	0.003
	Mean change at T2–T0 (LLM group)	−1.46 (-1.83,−1.08)	< 0.001
	Difference in mean change at T1–T0 (LLM vs Control)	−0.25 (-0.84, 0.34)	0.404
	Difference in mean change at T2–T0 (LLM vs Control)	−0.58 (-1.14,−0.03)	0.040
HADS-D	Baseline difference between groups (LLM vs Control)	−0.07 (-0.48, 0.34)	0.737
	Mean change at T1–T0 (Control group)	−0.64 (-1.07,−0.22)	0.003
	Mean change at T2–T0 (Control group)	−0.74 (-1.12,−0.35)	< 0.001
	Mean change at T1–T0 (LLM group)	−1.35 (-1.72,−0.99)	< 0.001
	Mean change at T2–T0 (LLM group)	−1.31 (-1.67,−0.96)	< 0.001
	Difference in mean change at T1–T0 (LLM vs Control)	−0.71 (-1.27,−0.15)	0.013
	Difference in mean change at T2–T0 (LLM vs Control)	−0.57 (-1.10,−0.05)	0.032
PSS-10	Baseline difference between groups (LLM vs Control)	0.06 (-0.73, 0.84)	0.884
	Mean change at T1–T0 (Control group)	−0.93 (-1.71,−0.14)	0.021
	Mean change at T2–T0 (Control group)	−1.40 (-2.15,−0.66)	< 0.001
	Mean change at T1–T0 (LLM group)	−1.97 (-2.73,−1.22)	< 0.001
	Mean change at T2–T0 (LLM group)	−2.65 (-3.40,−1.91)	< 0.001
	Difference in mean change at T1–T0 (LLM vs Control)	−1.05 (-2.14, 0.04)	0.059
	Difference in mean change at T2–T0 (LLM vs Control)	−1.25 (-2.31,−0.20)	0.020
MSPSS	Baseline difference between groups (LLM vs Control)	−0.05 (-1.33, 1.22)	0.937
	Mean change at T1–T0 (Control group)	0.13 (-1.13, 1.40)	0.835
	Mean change at T2–T0 (Control group)	0.52 (-0.69, 1.73)	0.397
	Mean change at T1–T0 (LLM group)	1.14 (-0.07, 2.35)	0.065
	Mean change at T2–T0 (LLM group)	1.88 (0.80, 2.97)	< 0.001
	Difference in mean change at T1–T0 (LLM vs Control)	1.01 (-0.74, 2.76)	0.259
	Difference in mean change at T2–T0 (LLM vs Control)	1.36 (-0.27, 2.99)	0.101
Caregiving self-efficacy	Baseline difference between groups (LLM vs Control)	0.59 (-0.02, 1.21)	0.057
	Mean change at T1–T0 (Control group)	2.27 (1.56, 2.97)	< 0.001
	Mean change at T2–T0 (Control group)	4.19 (3.50, 4.89)	< 0.001
	Mean change at T1–T0 (LLM group)	2.97 (2.38, 3.55)	< 0.001
	Mean change at T2–T0 (LLM group)	4.89 (4.18, 5.61)	< 0.001
	Difference in mean change at T1–T0 (LLM vs Control)	0.70 (-0.22, 1.61)	0.135
	Difference in mean change at T2–T0 (LLM vs Control)	0.70 (-0.30, 1.69)	0.171
WHOQOL physical	Baseline difference between groups (LLM vs Control)	0.78 (-2.20, 3.76)	0.609
	Mean change at T1–T0 (Control group)	4.85 (1.72, 7.98)	0.002
	Mean change at T2–T0 (Control group)	8.82 (5.63, 12.01)	< 0.001
	Mean change at T1–T0 (LLM group)	7.50 (4.66, 10.34)	< 0.001
	Mean change at T2–T0 (LLM group)	10.80 (7.69, 13.91)	< 0.001
	Difference in mean change at T1–T0 (LLM vs Control)	2.65 (-1.58, 6.88)	0.219
	Difference in mean change at T2–T0 (LLM vs Control)	1.98 (-2.48, 6.44)	0.384
WHOQOL psychological	Baseline difference between groups (LLM vs Control)	−0.49 (-3.72, 2.74)	0.767
	Mean change at T1–T0 (Control group)	4.13 (1.28, 6.99)	0.005
	Mean change at T2–T0 (Control group)	6.44 (3.58, 9.29)	< 0.001
	Mean change at T1–T0 (LLM group)	7.84 (4.62, 11.06)	< 0.001
	Mean change at T2–T0 (LLM group)	12.24 (9.16, 15.32)	< 0.001
	Difference in mean change at T1–T0 (LLM vs Control)	3.71 (-0.60, 8.01)	0.091
	Difference in mean change at T2–T0 (LLM vs Control)	5.80 (1.61, 10.00)	0.007
WHOQOL social	Baseline difference between groups (LLM vs Control)	1.34 (-2.00, 4.68)	0.431
	Mean change at T1–T0 (Control group)	2.07 (-1.08, 5.22)	0.198
	Mean change at T2–T0 (Control group)	1.75 (-1.27, 4.77)	0.257
	Mean change at T1–T0 (LLM group)	2.97 (-0.61, 6.55)	0.104
	Mean change at T2–T0 (LLM group)	2.10 (-1.47, 5.67)	0.248
	Difference in mean change at T1–T0 (LLM vs Control)	0.90 (-3.86, 5.67)	0.710
	Difference in mean change at T2–T0 (LLM vs Control)	0.35 (-4.32, 5.03)	0.882
WHOQOL environmental	Baseline difference between groups (LLM vs Control)	−0.46 (-2.54, 1.63)	0.668
	Mean change at T1–T0 (Control group)	0.82 (-1.21, 2.85)	0.428
	Mean change at T2–T0 (Control group)	2.66 (0.57, 4.75)	0.013
	Mean change at T1–T0 (LLM group)	3.56 (1.49, 5.64)	< 0.001
	Mean change at T2–T0 (LLM group)	5.66 (3.44, 7.89)	< 0.001
	Difference in mean change at T1–T0 (LLM vs Control)	2.74 (-0.15, 5.64)	0.064
	Difference in mean change at T2–T0 (LLM vs Control)	3.00 (-0.05, 6.05)	0.054

βadj indicates the adjusted coefficient estimated from the GEE model, and 95% CI indicates the 95% confidence interval. GEE, generalized estimating equations; LLM, large language model; ZBI, zarit burden interview; HADS-A, hospital anxiety and depression scale-anxiety subscale; HADS-D, hospital anxiety and depression scale-depression subscale; PSS-10, 10-item perceived stress scale; MSPSS, multidimensional scale of perceived social support; WHOQOL, world health organization quality of life.

### 3.5 Dose-response analysis

**Figure 2:**
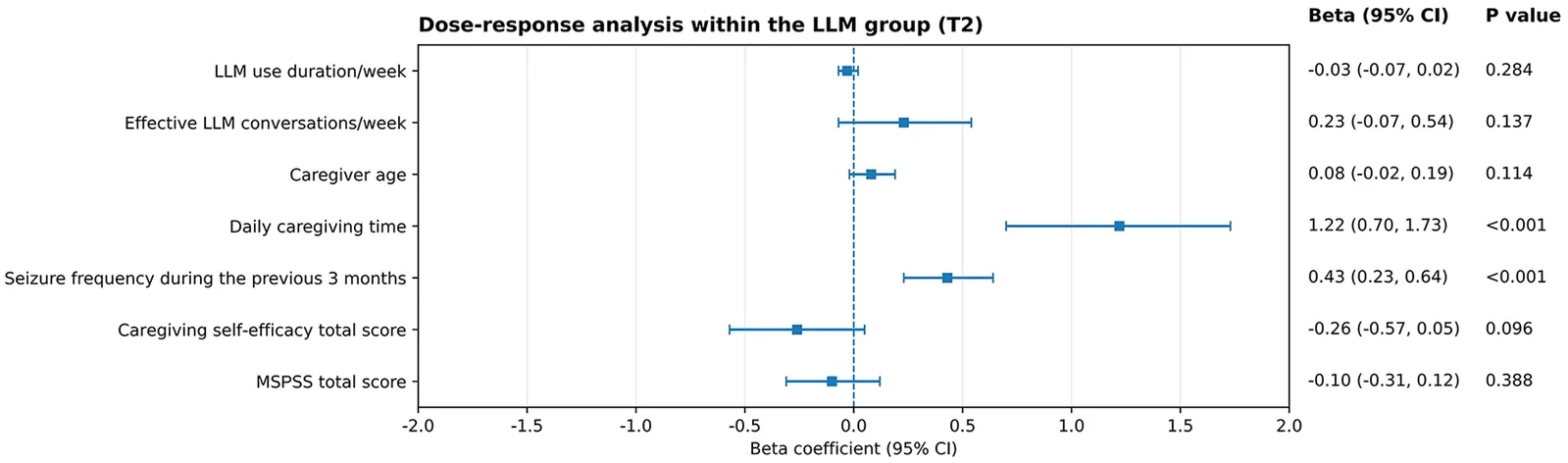
Forest plot. LLM = large language model; β = Beta coefficient; CI = confidence interval.

Within the LLM group, exploratory dose-response analyses showed no significant linear association between weekly use time and the 3-month ZBI total score, nor between the weekly number of effective conversations and the 3-month ZBI total score. Daily caregiving time and seizure frequency remained associated with caregiver burden in these models. Overall, no clear dose-response relationship was observed between the measured intensity of LLM use and caregiver burden during the 3-month follow-up period (Figure 2).

## 4 Discussion

In this prospective cohort study, 296 primary caregivers of patients with epilepsy were followed at baseline, 1 month, and 3 months. Under generally comparable baseline conditions, caregivers who used DeepSeek for epilepsy caregiving-related support showed greater reductions in caregiver burden during follow-up and more favorable changes in psychosocial outcomes, including anxiety, depression, perceived stress, social support, quality of life, and caregiving self-efficacy. Further GEE analyses showed that LLM use was significantly associated with longitudinal improvement in caregiver burden and emotional symptoms. After adjustment for multiple confounders, LLM use remained associated with a greater reduction in ZBI scores over 3 months. These findings suggest that, in epilepsy caregiving, LLMs may serve not only as information tools but also as a source of broader psychosocial support.

These findings are generally consistent with previous research on caregiver burden in epilepsy. Earlier studies have shown that caregivers of adults with epilepsy often experience substantial burden, which is associated with poorer quality of life, higher levels of anxiety and depression, and greater perceived stress. Recent reviews have further suggested that caregiver burden is jointly influenced by disease severity, caregiving intensity, family resources, and psychosocial factors (18, 19). In particular, caregiving time, seizure frequency, family support, and family functioning have repeatedly been identified as important correlates of caregiver burden (20). In the present study, the LLM group showed a faster and greater decline in ZBI scores during follow-up, and the adjusted GEE model showed significantly greater reductions in ZBI scores over time in the LLM group than in the control group, suggesting a longitudinal association between LLM use and lower caregiver burden after adjustment for prespecified baseline covariates.

Several mechanisms may explain how LLM use could influence caregiver burden and psychosocial outcomes in epilepsy. Epilepsy caregiving is long term and highly uncertain, and caregivers often need timely, understandable, and practical support for seizure monitoring, medication management, clinic preparation, and emotional care. Previous studies have shown that patients with epilepsy and their caregivers often have unmet information needs, while existing online epilepsy materials are frequently difficult to read and translate into practical caregiving guidance (4). In this context, the immediate response, interactivity, and individualized explanations provided by LLMs may help reduce uncertainty and decision-related stress. By translating complex medical information into more understandable language, LLMs may also improve caregivers' sense of control and self-efficacy, thereby indirectly reducing caregiver burden (8). In addition, interactive support itself may partly buffer the subjective experience of “coping alone,” which may help explain the concurrent improvement observed in perceived social support and quality of life.

This interpretation is also in line with the broader literature on AI and LLMs in caregiving. Systematic reviews suggest that, although still at an early stage, AI-based tools for informal caregivers have shown potential in burden identification, remote monitoring, decision support, and care coordination (21, 22). Reviews of conversational LLMs in healthcare indicate that their most established strengths lie in information summarization, medical question answering, and information collection, although concerns remain regarding reliability, consistency, bias, and privacy. In addition, recent studies in family caregiving settings suggest that LLMs may have value in improving clarity, specificity, and self-efficacy, even though their performance has not yet reached the level of professional “gold standard” support (23). Taken together, these findings provide a theoretical basis for the present results and suggest that LLMs may have promising transferability to epilepsy caregiving settings.

Notably, within the LLM group, neither weekly use time nor weekly number of effective conversations showed a significant linear association with the 3-month ZBI score. This suggests that the potential benefit of LLM use may depend less on how much the tool is used and more on whether it is used regularly, whether effective support is obtained in key caregiving situations, and whether the interaction quality is sufficient. Previous research in family caregiving has similarly suggested that the value of LLMs may depend more on clarity, specificity, contextual relevance, and their ability to enhance self-efficacy than on the number of interactions alone (10, 24). Therefore, the absence of a clear dose-response relationship in this study does not necessarily mean that usage frequency is unimportant. Rather, it may reflect a threshold effect, the limited ability of crude frequency measures to capture actual quality of use, or limitations of self-reported exposure assessment.

From a practical perspective, our findings suggest that LLMs may serve as a useful complement, rather than a replacement, in epilepsy family caregiving. For caregivers who must continuously cope with seizure unpredictability, repeated caregiving questions, and ongoing psychological stress, the low-threshold, immediate feedback, and repeatable interaction offered by LLMs may make them an important source of support beyond routine outpatient education, especially in settings with limited specialist resources, limited opportunities for face-to-face consultation, or delayed access to professional help (11, 25). At the same time, the boundaries of their use must be clearly recognized. Previous studies have shown that even high-performing LLMs may omit key steps or oversimplify complex tasks and therefore cannot replace professional guidance. In epilepsy care, LLMs may be most appropriate for information support, communication assistance, and preliminary problem clarification, whereas medication adjustment, emergency management, and other high-risk clinical decisions should still be supervised by neurology professionals (26, 27).

Several limitations should be noted. First, this was a prospective cohort study rather than a randomized controlled trial. Although the two groups were generally comparable at baseline and multiple confounders were adjusted for, residual confounding and selection bias cannot be ruled out, and the findings should not be interpreted as causal. Second, LLM exposure was partly based on structured self-report when platform logs were unavailable, while all primary and secondary outcomes were self-reported. Although validated questionnaires were used, recall bias and social desirability bias may have affected the accuracy of the reported outcomes and partly explain the lack of a clear dose-response relationship. Third, the follow-up period was limited to 3 months, so only short- and medium-term associations could be assessed. Fourth, this study evaluated real-world LLM use for epilepsy caregiving support but did not independently review each output, and therefore could not directly assess the accuracy or safety of DeepSeek in specific caregiving situations. Existing studies have also highlighted reliability, consistency, bias, and privacy as important concerns in the use of LLMs in healthcare.

Overall, this study suggests that, over 3 months, the use of DeepSeek for epilepsy caregiving-related support was associated with lower caregiver burden and better psychosocial outcomes, especially anxiety, depression, and perceived stress. This association remained significant after adjustment for multiple potential confounders. These findings are consistent with previous studies on caregiver burden in epilepsy, unmet information needs, and the potential role of AI and LLMs in caregiving support. They also suggest that LLMs may serve as a low-threshold and scalable supportive tool in epilepsy family caregiving. However, further rigorous real-world studies are still needed to evaluate their long-term effectiveness, safety, and appropriate scope of use.

## Data Availability

The raw data supporting the conclusions of this article will be made available by the authors, without undue reservation.
