# Supplementary material for: Large language model–assisted support among caregivers of patients with epilepsy: associations with caregiver burden and psychosocial outcomes
Source: Front Neurol. 2026 Sep 9;17:1912114. doi: 10.3389/fneur.2026.1912114 (PMC13598279; doi:10.3389/fneur.2026.1912114)
Supplement: Supplementary file 1 [file Table_1.docx]

**Supplementary Table S1. Distribution of severity and interpretive categories for ZBI, HADS-A, HADS-D, and MSPSS**

| **Outcome** | **Time point** | **Severity category** | **Overall (N=296)** | **Control group (n=149)** | **LLM group (n=147)** | **P value** |
| --- | --- | --- | --- | --- | --- | --- |
| ZBI total score | T0 baseline | Little or no burden (0–20) | 37 (12.5%) | 14 (9.4%) | 23 (15.6%) | 0.202 |
|  |  | Mild to moderate burden (21–40) | 221 (74.7%) | 113 (75.8%) | 108 (73.5%) |  |
|  |  | Moderate to severe burden (41–60) | 38 (12.8%) | 22 (14.8%) | 16 (10.9%) |  |
|  |  | Severe burden (61–88) | 0 (0.0%) | 0 (0.0%) | 0 (0.0%) |  |
|  | T1 1 month | Little or no burden (0–20) | 88 (29.7%) | 37 (24.8%) | 51 (34.7%) | 0.114 |
|  |  | Mild to moderate burden (21–40) | 196 (66.2%) | 104 (69.8%) | 92 (62.6%) |  |
|  |  | Moderate to severe burden (41–60) | 12 (4.1%) | 8 (5.4%) | 4 (2.7%) |  |
|  |  | Severe burden (61–88) | 0 (0.0%) | 0 (0.0%) | 0 (0.0%) |  |
|  | T2 3 months | Little or no burden (0–20) | 118 (39.9%) | 51 (34.2%) | 67 (45.6%) | 0.059 |
|  |  | Mild to moderate burden (21–40) | 172 (58.1%) | 93 (62.4%) | 79 (53.7%) |  |
|  |  | Moderate to severe burden (41–60) | 6 (2.0%) | 5 (3.4%) | 1 (0.7%) |  |
|  |  | Severe burden (61–88) | 0 (0.0%) | 0 (0.0%) | 0 (0.0%) |  |
| HADS-A | T0 baseline | Non-case (0–7) | 201 (67.9%) | 97 (65.1%) | 104 (70.7%) | 0.578 |
|  |  | Doubtful case (8–10) | 85 (28.7%) | 46 (30.9%) | 39 (26.5%) |  |
|  |  | Definite case (11–21) | 10 (3.4%) | 6 (4.0%) | 4 (2.7%) |  |
|  | T1 1 month | Non-case (0–7) | 224 (75.7%) | 109 (73.2%) | 115 (78.2%) | 0.442 |
|  |  | Doubtful case (8–10) | 63 (21.3%) | 36 (24.2%) | 27 (18.4%) |  |
|  |  | Definite case (11–21) | 9 (3.0%) | 4 (2.7%) | 5 (3.4%) |  |
|  | T2 3 months | Non-case (0–7) | 241 (81.4%) | 116 (77.9%) | 125 (85.0%) | 0.253 |
|  |  | Doubtful case (8–10) | 51 (17.2%) | 30 (20.1%) | 21 (14.3%) |  |
|  |  | Definite case (11–21) | 4 (1.4%) | 3 (2.0%) | 1 (0.7%) |  |
| HADS-D | T0 baseline | Non-case (0–7) | 239 (80.7%) | 120 (80.5%) | 119 (81.0%) | 0.760 |
|  |  | Doubtful case (8–10) | 53 (17.9%) | 26 (17.4%) | 27 (18.4%) |  |
|  |  | Definite case (11–21) | 4 (1.4%) | 3 (2.0%) | 1 (0.7%) |  |
|  | T1 1 month | Non-case (0–7) | 270 (91.2%) | 134 (89.9%) | 136 (92.5%) | 0.539 |
|  |  | Doubtful case (8–10) | 26 (8.8%) | 15 (10.1%) | 11 (7.5%) |  |
|  |  | Definite case (11–21) | 0 (0.0%) | 0 (0.0%) | 0 (0.0%) |  |
|  | T2 3 months | Non-case (0–7) | 277 (93.6%) | 136 (91.3%) | 141 (95.9%) | 0.086 |
|  |  | Doubtful case (8–10) | 18 (6.1%) | 13 (8.7%) | 5 (3.4%) |  |
|  |  | Definite case (11–21) | 1 (0.3%) | 0 (0.0%) | 1 (0.7%) |  |
| MSPSS total score | T0 baseline | Low perceived support (12–35) | 3 (1.0%) | 2 (1.3%) | 1 (0.7%) | 1.000 |
|  |  | Moderate perceived support (36–60) | 283 (95.6%) | 142 (95.3%) | 141 (95.9%) |  |
|  |  | High perceived support (61–84) | 10 (3.4%) | 5 (3.4%) | 5 (3.4%) |  |
|  | T1 1 month | Low perceived support (12–35) | 2 (0.7%) | 1 (0.7%) | 1 (0.7%) | 0.904 |
|  |  | Moderate perceived support (36–60) | 276 (93.2%) | 138 (92.6%) | 138 (93.9%) |  |
|  |  | High perceived support (61–84) | 18 (6.1%) | 10 (6.7%) | 8 (5.4%) |  |
|  | T2 3 months | Low perceived support (12–35) | 1 (0.3%) | 1 (0.7%) | 0 (0.0%) | 0.222 |
|  |  | Moderate perceived support (36–60) | 278 (93.9%) | 142 (95.3%) | 136 (92.5%) |  |
|  |  | High perceived support (61–84) | 17 (5.7%) | 6 (4.0%) | 11 (7.5%) |  |

Note: Data are presented as n (%).

ZBI = Zarit Burden Interview; HADS-A = Hospital Anxiety and Depression Scale-Anxiety subscale; HADS-D = Hospital Anxiety and Depression Scale-Depression subscale; MSPSS = Multidimensional Scale of Perceived Social Support.
